# *HRAS* mutation positive multiple myeloma in the type 2 *CALR* mutation positive essential thrombocythemia: A case report

**DOI:** 10.1111/jcmm.17647

**Published:** 2023-01-05

**Authors:** Lewandowski Krzysztof, Kopydłowska Agata, Kanduła Zuzanna, Bartłomiej Sankowski, Marcin Machnicki, Barańska Marta, Gwóźdź‐Bąk Kinga, Kubicki Tadeusz, Płotka Anna, Przysiecka Łucja, Dworacki Grzegorz, Kozłowski Piotr, Stokłosa Tomasz

**Affiliations:** ^1^ Department of Hematology and Bone Marrow Transplantation Poznań University of Medical Sciences Poznań Poland; ^2^ Department of Tumor Biology and Genetics Medical University of Warsaw Warsaw Poland; ^3^ NanoBioMedical Centre Adam Mickiewicz University in Poznań Poznań Poland; ^4^ Department of Clinical Pathology Poznań University of Medical Sciences Poznań Poland; ^5^ Laboratory of Genomics Institute of Bioorganic Chemistry, Polish Academy of Sciences Poznan Poland

**Keywords:** CALR, essential thrombocythemia, HRAS, multiple myeloma

## Abstract

Out of *BCR‐ABL* negative myeloproliferative neoplasm (MPNPh^−^) patients, 3%–14% display a concomitant monoclonal gammopathy of unknown significance (MGUS). In most cases, the diagnosis of plasma cell dyscrasia is either synchronous with that of MPNPh^−^ or occurs later on. We present a 50‐year‐old patient with type 2 *CALR* Lys385Asnfs*47 mutation positive essential thrombocythemia (ET) who developed symptomatic multiple myeloma (MM) 13 years after the diagnosis of ET during PEG‐INF2α treatment. The NGS study performed at the time of the MM diagnosis revealed the HRAS Val14Gly/c.41T〉G mutation and the wild type *CALR*, JAK2 and MPL gene sequence. In the presented case, the complete molecular remission of ET was achieved after 16 months of PEG‐INF2α treatment. The origin of MM cells in MPNPh^−^ patients remains unknown. Published data suggests that type 2 CALRins5 up‐regulate the ATF6 chaperone targets in hematopoietic cells and activate the inositol‐requiring enzyme 1α‐X‐box‐binding protein 1 pathway of the unfolded protein response (UPR) system to drive malignancy. It cannot be excluded that endoplasmic reticulum stress induced by the increased ATF6 resulted in an abnormal redox homeostasis and proteostasis, which are factors linked to MM. The presented case history and the proposed mechanism of mutant *CALR* interaction with UPR and/or ATF6 should initiate the discussion about the possible impact of the mutant *CALR* protein on the function and genomic stability of different types of myeloid cells, including progenitor cells.

Out of *BCR‐ABL* negative myeloproliferative neoplasm (MPNPh^−^) patients, 3%–14% display a concomitant monoclonal gammopathy (MGUS).^1^, ^2^ The diagnosis of MPN proceeds lymphoproliferative disease (LPD) occurrence in about 50% of patients. The median time between the diagnosis of MPNPh^−^ and LPD was established at 72 months.^3^ Herein, we present an unusual outcome of low risk essential thrombocythemia (ET), diagnosed in 2008 in a 50‐year‐old woman. Initially, she was treated with hydroxyurea (HC) 1.5 g/daily orally. Her medical history revealed mitral and aortal valve insufficiency, which was diagnosed in 2018, and the episode of transient ischaemic attack (2018), resulting in foci cerebral ischaemia (MRI). In 2019, due to toxicity, the HC treatment was stopped. The therapy with the pegylated interferonα 2a (*Pegasys*, PEG‐INFα2a) was started in August 2020. The molecular work‐up showed no *JAK2* V617F and *MPL* exon 10 mutation and the presence of the type 2 *CALR* mutation‐ LRG_828t1:c.1154_1155insTTGTC, LRG_828p1:p.(Lys385Asnfs*47) (VAF 35%). The bone marrow (BM) biopsy performed in 2020 showed the normocellular BM, locally hypercellular, with an erythroid/granulocytic cell ratio of 1:3, and normal erythroid and granulocytic proliferation index and maturation pattern. The blast cell (CD34^+^, CD117^+^) content was determined at 1%–2% of nuclear cells. An increased number of medium‐sized and large megakaryocytes (factor VIII^+^) was found, without the tendency to form clusters (locally forming loose clusters consisting of 4–8 cells). The BM fibrosis (MF) grade according to the European consensus criteria was 0/1.

In September 2021, she experienced a fracture of the L1 vertebrae of the spine which was treated with Th12‐L1 stabilization with laminectomy. The PET‐CT scan revealed multiple osteolytic lesions in the bones. Immunofixation studies showed the IgG kappa monoclonal protein in the blood at the concentration of 49.6 g/L. The WBS performed in January 2022 documented the fracture of Th9, the presence of an intramedullary tumour in the bottom part of the stern. The MFC of the BM cells showed 65% of abnormal plasma cells. The repeated biopsy documented BM cellularity 60% with about 30% of dispersed plasma cells, locally forming infiltrates. Erythroid cell line content e‐cadherin^+^ was determined at 30%. The granulocytic cell [CD15^+^, MPO^+^] line and megakaryocyte (factor VIII^+^, CD61^+^) evaluation showed a normal morphology and maturation pattern. MF +1. Retrospective analysis of a biopsy sample collected in 2020 (before PEG‐INFα2a treatment initiation) confirmed the presence of a small, abnormal plasma cell population with the same immunophenotypic characteristic (Figure 1).

**FIGURE 1:**
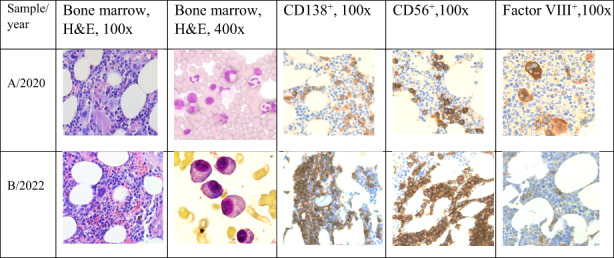
The bone marrow (BM) trephine biopsy sample histopathological evaluation results in type 2 *CALR* mutation positive essential thrombocythemia patient developing multiple myeloma during disease outcome. Multiple myeloma cells immunophenotype: CD138^++^, MUM1^+^, CD56, CD20^−^, cyclin D1^−^, Ig‐CISH‐kappa^+++^/Ig‐lambda‐.

An NGS analysis of the BM cells was performed using a sample collected in January 2022, before MM treatment initiation. The targeted enrichment approach with a custom‐designed gene panel revealed the presence of the Harvey rat sarcoma (*HRAS*) gene mutation (chr11:000534282‐A > C VAF 14% NM_001130442.1:p.Val14Gly/c.41 T > G) and the wild type of *CALR*, *JAK2* and *MPL* gene sequence (Figure S1). The analysis of the blood sample collected at the time of PEG‐INF2α treatment initiation using the SS did not confirm the presence of the *HRAS* variant. The *HRAS* mutations were also undetectable in the DNA obtained from the blood MNC and buccal swab cells collected immediately before MM treatment initiation (Figure S1).

After the diagnosis of MM, the treatment with bortezomib, thalidomide and dexamethasone (VTD) was introduced. After three VTD therapy cycles, a significant reduction of the protein M concentration in the blood from 49.6 g/L to 3.6 g/L was confirmed (Figure 2).

**FIGURE 2:**
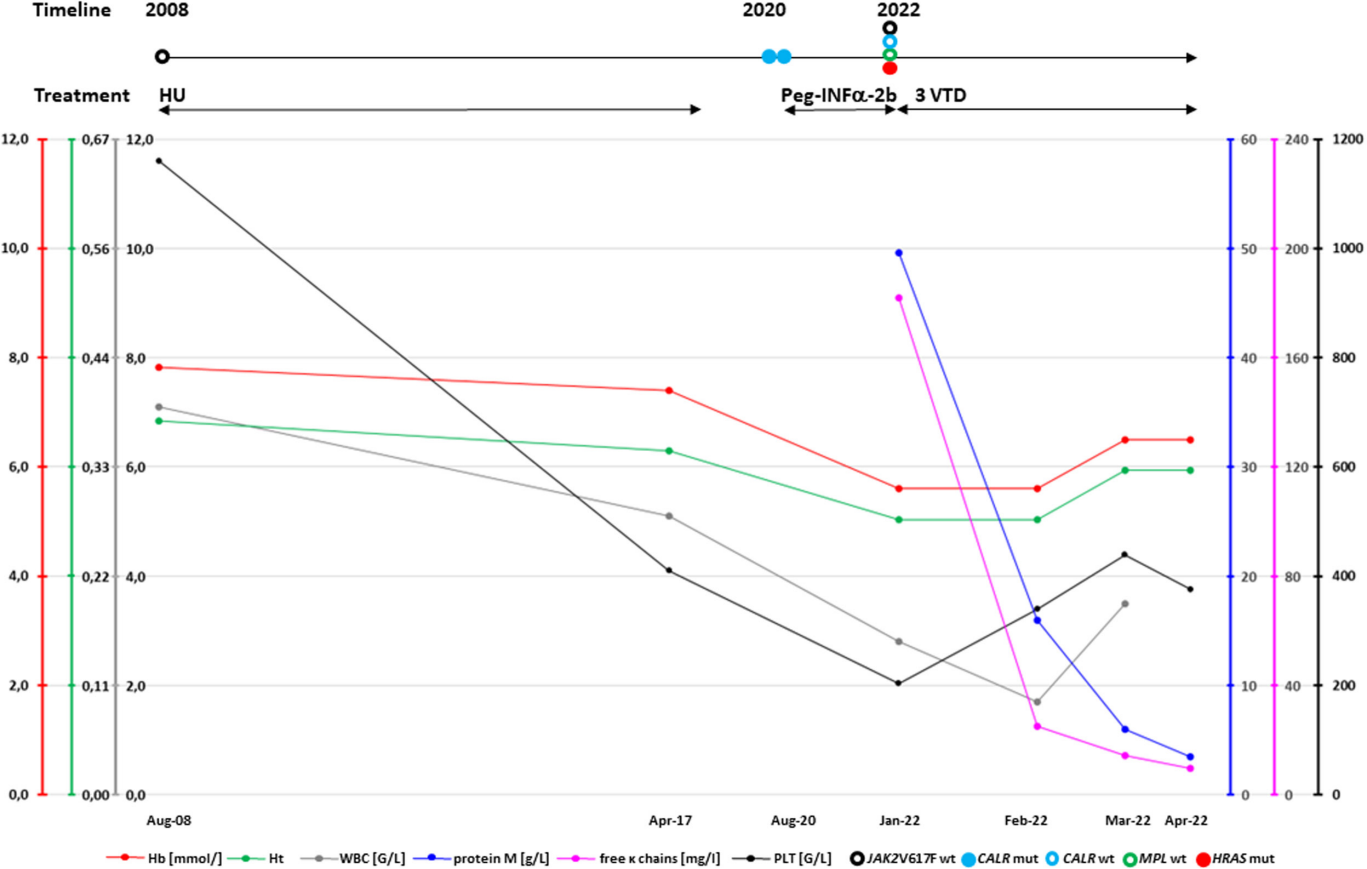
The changes in the basic laboratory parameters in a patient with type 2 *CALR* mutation positive essential thrombocythemia developing multiple myeloma during disease outcome. The timeline and treatment type are shown.

In the presented case, the mechanism of the *CALR* mutation positive clone disappearance is not clear. It cannot be ruled out that the PEG‐INFα2a treatment resulted in HSC exhaustion and *CALR* mutation positive clone eradication. The mentioned hypothesis is in agreement with the published data confirming that INFα treatment resulted in complete molecular remission (CMR) in 10% of ET patients.^4^ It is probable that PEG‐INFα targets the type 2 *CALR* mutation positive haematopoietic stem and progenitor cells preferentially, as was documented by Mosca et al.^5^

The published data suggest that the CALRins5 (type 2) in comparison to the CALRdel52 (type 1) differentially activate the inositol‐requiring enzyme 1α (IRE1α)‐X‐box‐binding protein 1 (XBP1) pathway of the unfolded protein response system to drive malignancy. The up‐regulation of the ATF6 chaperone targets specifically in CALRins5 cells suggests that the loss of the chaperone function by CALRins5 may underlie the differential activation of ATF6.^6^ In 2019, Salati et al. demonstrated that *CALR* mutations induce increased sensitivity to oxidative stress, leading to the increase of oxidative DNA damage and the down‐modulation of the oxidation resistance gene 1 in *CALR*‐mutated cells.^7^ The experimental study of bone marrow cells confirmed the presence of the CALR mutation even in multipotent precursors (MPP) of lymphohematopoiesis.^8^ Therefore, it is possible that the RE stress due to the CALRins5 in MPP promotes oncogenesis via the abnormal function of IRE1a‐XBP1 axis, even in MPP. Another probable mechanism responsible for secondary malignancies development in MPNPh‐ includes immunosuppressive effects mediated by exon‐9‐mutated CALR released from the malignant cell, resulting in the inhibition of phagocytosis of dying cancer cells by dendritic cells.^9^ The above‐mentioned data suggest a possible association between the *CALR* mutation driven malignancies and an increased risk of secondary neoplasms, including MM. In the systematic review done by Marchetti et al., the coexistence of the type 1 *CALR*del52 mutation positive ET and MGUS was reported in 3 patients.^3^ Loscocco et al. described a *CALR*del52 mutation positive (VAF 57%) ET case who was diagnosed with smouldering MM 1 year after the diagnosis of ET. Four years later, the progression to post‐ET myelofibrosis was documented. After another 2 years, the MGUS evolution to the IgG lambda MM was observed.^10^

The occurrence of MM is rare in JAK2 mutation positive PV patients and no case of an MM diagnosis preceding an MPN one has been reported. It was shown that 9% of patients with MPN (36.0% of ET) harboured an M‐protein. ET‐MGUS (IgGκ and IgMκ) was diagnosed in 20% of ET patients, including cases with CALR type 1 and type 2 mutations.^11^ The frequency of the coexistence of MM with ET was established at 15%.^3^ The mentioned data are in agreement with a previous report documenting the direct role of calnexin, calreticulin, and tapasin abnormalities in the MGUS progression to MM.^12^

The presented case is the first reported *HRAS* Val14Gly/c.41 T > G variant positive haematological malignancy. The *HRAS* variant detected in our patient had been reported only once in Clinvar (accession number VCV000654373.1) in the case of Costello Syndrome. It is likely pathogenic according to Clinvar and of uncertain significance according to Varsome. The Functional Analysis Through Hidden Markov Models (FATHMM‐MKL) categorizes this change as damaging with a 0.99 coding score. Algorithms developed to predict the effect of missense changes on protein structure and function (SIFT, PolyPhen‐2, Align‐GVGD) all suggest that this variant is likely to be disruptive, but these predictions have not been confirmed by published functional studies (Clinvar).

The presence of *NRAS* and *KRAS* mutations was confirmed in 8% and 4% of myeloid malignancies. It was documented that missense mutations at codons 12 and 13 are thought to limit GTPase‐activating protein (GAP) interaction with the GTPase site of RAS proteins, preventing their hydrolysis to an inactive state. It was previously documented that mutations of RAS family genes contribute to myelomagenesis, including the transformation from MGUS to MM.^13^ In MM patients, the presence of the *NRAS* and *KRAS* gene mutations was confirmed in 19.5% and 24.2% of patients, respectively.^14^ The data concerning *HRAS* mutations in myeloid disorders are limited. The role of *HRAS* gene mutations in the process of the malignant transformation of plasma cells also remains unknown. In the report of O'Donnell et al., a *HRAS* mutation [c.181 C > A (p.Q61K)] was identified in 1/67 of patients with MM.^15^ Frontzek et al.^16^ confirmed the presence of *HRAS* mutations in 2% of patients with plasmablastic lymphoma.

One still unsolved mystery is that of the origin of MM cells in patients with MPNPh‐. The available data suggest that hydroxyurea or interferonα treatments do not increase the risk of secondary cancers. In the summary of the published case reports and studies prepared by Malhotra et al., it has not yet been definitively shown that these 2 entities arise from a common‐ancestor HSPC.^1^ In 2021, Hui W. et al. identified eight proteins that were found to be dysregulated differently in ET patients with mutated CALRdel52 and those with JAK2V617F mutation. The proteomic analysis showed that 20 proteins were altered in ET with the CALRdel52 mutation: those involved in cell signalling (Erk1/2, PTEN, Raf‐B, Rap1, Axin, ERβ, TGF‐β), cell cycle (Cdc42, Cdc2, CyclinD1, p27), apoptosis (Bcl‐Xl, c‐IAP2, NFκB p50, cPKCα, Survivin), transcription factor (eIF4B, SRC‐1), adhesion (E‐cadherin) and DNA repair (TDP1).^17^ Similar data, concerning CALR type 2 mutation detected in our patient is not available yet. Therefore, we performed an additional review of The Cancer Genome Atlas (TCGA) database, including datasets of >10,000 samples of 33 cancer types, searching for a possible co‐occurrence of the CALR driver mutations and mutations in the RAS family genes (HRAS, NRAS, and KRAS). We did not find any sample with co‐occurring CALR type 1 or type 2 mutations and mutations in the RAS family genes. The only few CALR mutations co‐occurring with mutations in the RAS genes seem to be random, they were mostly missense mutations randomly distributed in different positions of the CALR gene, detected mostly in cancers (UCEC and COAD) known to be frequently hypermutated due to different mutagenic processes and aberrations in DNA repair machinery (Table S1). We realize that our hypotheses have considerable limitations, as more studies (including functional studies) are necessary to explore the link between the CALR‐driven malignancies and secondary cancer development and origin. For this reason, the precise mechanism responsible for CALRmut‐driven oncogenesis remains unknown and is probably multifactorial.

The question is that of the medical significance of the presented data. In our opinion, more attention should be paid to the monitoring of MPN patients for the presence of MGUS, to allow for an early diagnosis of MM and the initiation of an appropriate treatment.^10^ Still, the study of the genetic aberration profile in MPNPh‐patients with coexisting MM is needed to precisely define and attack common molecular target(s) responsible for oncogenesis.^18^

## AUTHOR CONTRIBUTIONS

**Agata Lehmann‐Kopydłowska:** Data curation (equal); resources (equal); writing – review and editing (supporting). **Zuzanna Kanduła:** Data curation (equal); formal analysis (equal); investigation (equal); methodology (equal); visualization (equal); writing – review and editing (equal). **Bartłomiej Sankowski:** Investigation (equal); writing – review and editing (equal). **Marcin Machnicki:** Investigation (equal); writing – review and editing (equal). **Marta Barańska:** Data curation (equal); writing – review and editing (equal). **Kinga Gwóźdź‐Bąk:** Data curation (equal); investigation (equal); writing – review and editing (equal). **Tadeusz Kubicki:** Data curation (equal); methodology (equal); writing – review and editing (equal). **Anna Płotka:** Data curation (equal); investigation (supporting); writing – review and editing (equal). **Łucja Przysiecka:** Investigation (equal); resources (equal); writing – review and editing (equal). **Grzegorz Dworacki:** Investigation (equal); writing – original draft (supporting); writing – review and editing (equal). **T. Stoklosa:** Investigation (equal); methodology (equal); resources (equal); writing – original draft (supporting); writing – review and editing (equal). **Piotr Kozlowski:** Formal analysis (supporting); validation (supporting); writing – review and editing (supporting).

## CONFLICT OF INTEREST

The authors declare no conflict of interest.

## INFORMED CONSENT

The study was approved by the Bioethics Committee of the Poznan University of Medical Sciences, Poland (nos. 1056/16, 181/18, 846/21) and was conducted in accordance with the Declaration of Helsinki. Informed consent was obtained from the patient enrolled in this study. The samples were anonymised and then analysed.

## Supporting information


FigureS1

TableS1

## Data Availability

Not applicable.
