# Supplementary material for: HRAS mutation positive multiple myeloma in the type 2 CALR mutation positive essential thrombocythemia: A case report
Source: J Cell Mol Med. 2023 Jan 5;27(2):299–303. doi: 10.1111/jcmm.17647 (PMC9843526; doi:10.1111/jcmm.17647)
Supplement: Supplementary file 1 — FigureS1 [file JCMM-27-299-s002.docx]

Supplementary Figure 1. Results of Sanger and next generation sequencing of peripheral blood, buccal swap and bone marrow samples in patient with the type 2 calreticulin gene mutation positive essential thrombocythemia who developed the Harvey rat sarcoma gene mutation positive multiple myeloma during PEG-INF2α treatment.

The results of Sanger sequencing, high resolution heat melting analysis of *CALR* exon 9 and *HRAS* exon 1 are shown in A, B and C section, respectively. The results of NGS study of *HRAS* (exon1), JAK2 (exon 14), *CALR* exon 9 and *MPL* (exon 10) od the bone marrow sample collected at the time of multiple myeloma (MM) diagnosis, before MM treatment initiation are shown in the section D.

| A. `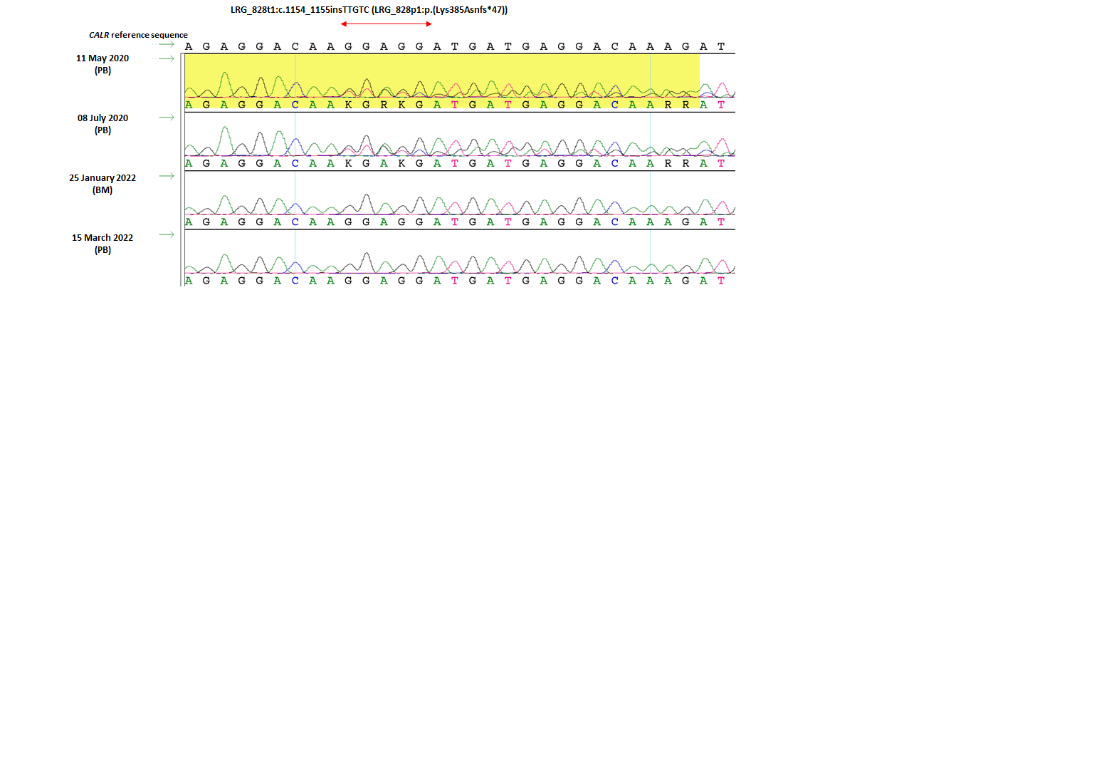 | C.  `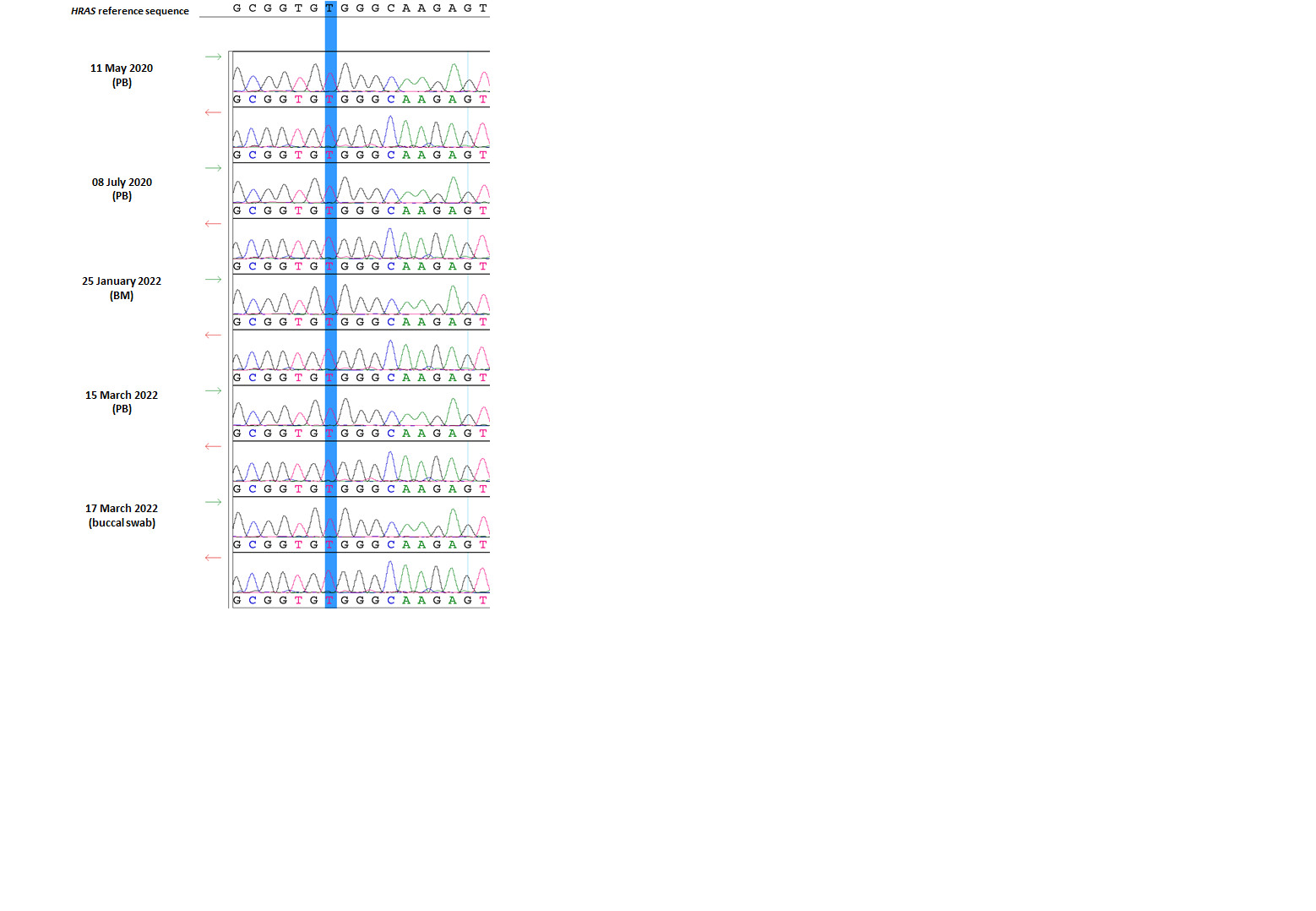     \| **Gene** \| **Protein change**  **(Coding change)** \| **VAF** \| **Cosmic ID** \| **NCBI Reference** \| \| --- \| --- \| --- \| --- \| --- \| \| **HRAS** \| Val14Gly  (c.41T>G) \| 14% \| COSM238586 \| NM_001130442.1 \| |
| --- | --- | --- | --- | --- | --- | --- | --- | --- | --- | --- | --- |
| B `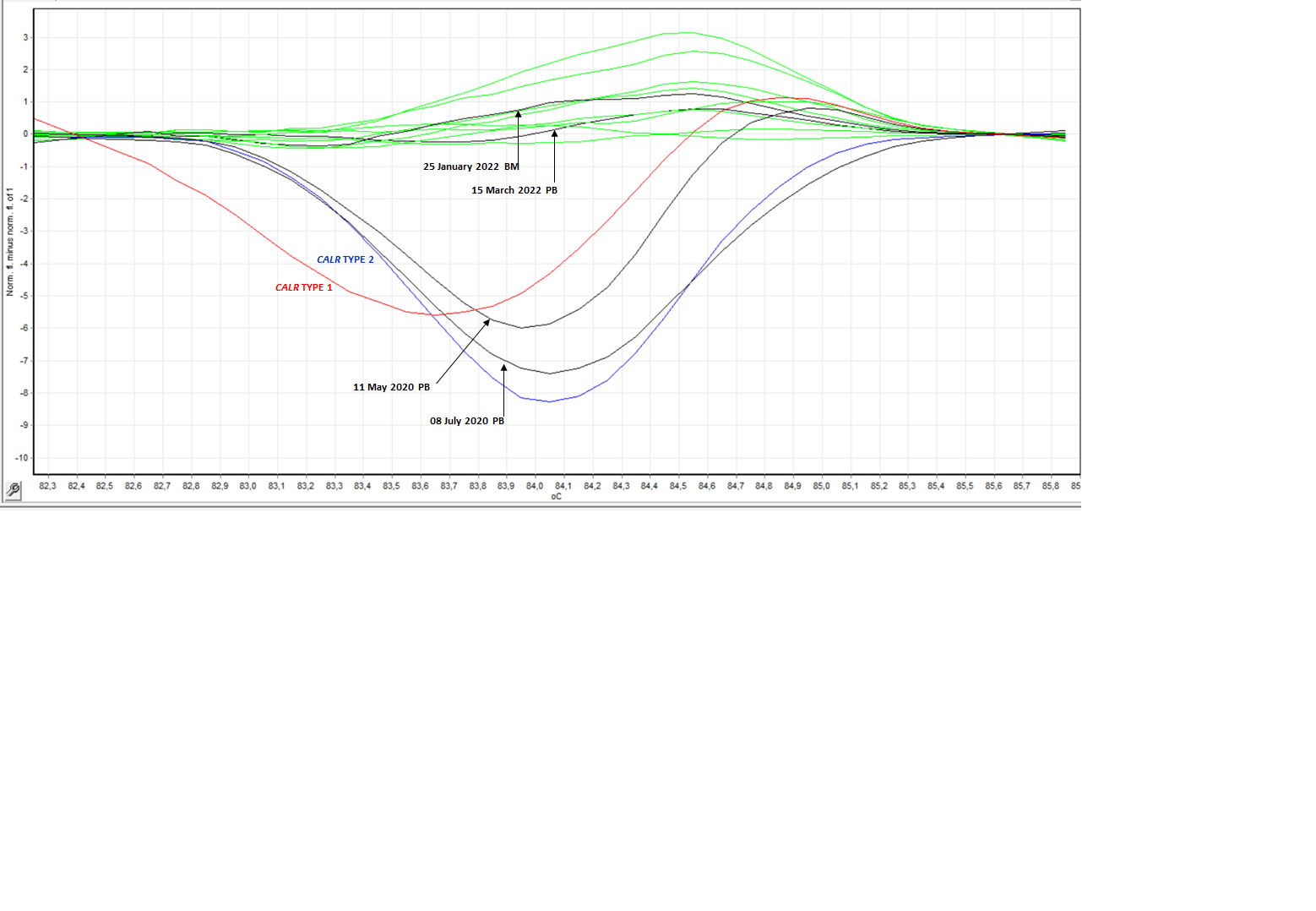 |  |

D


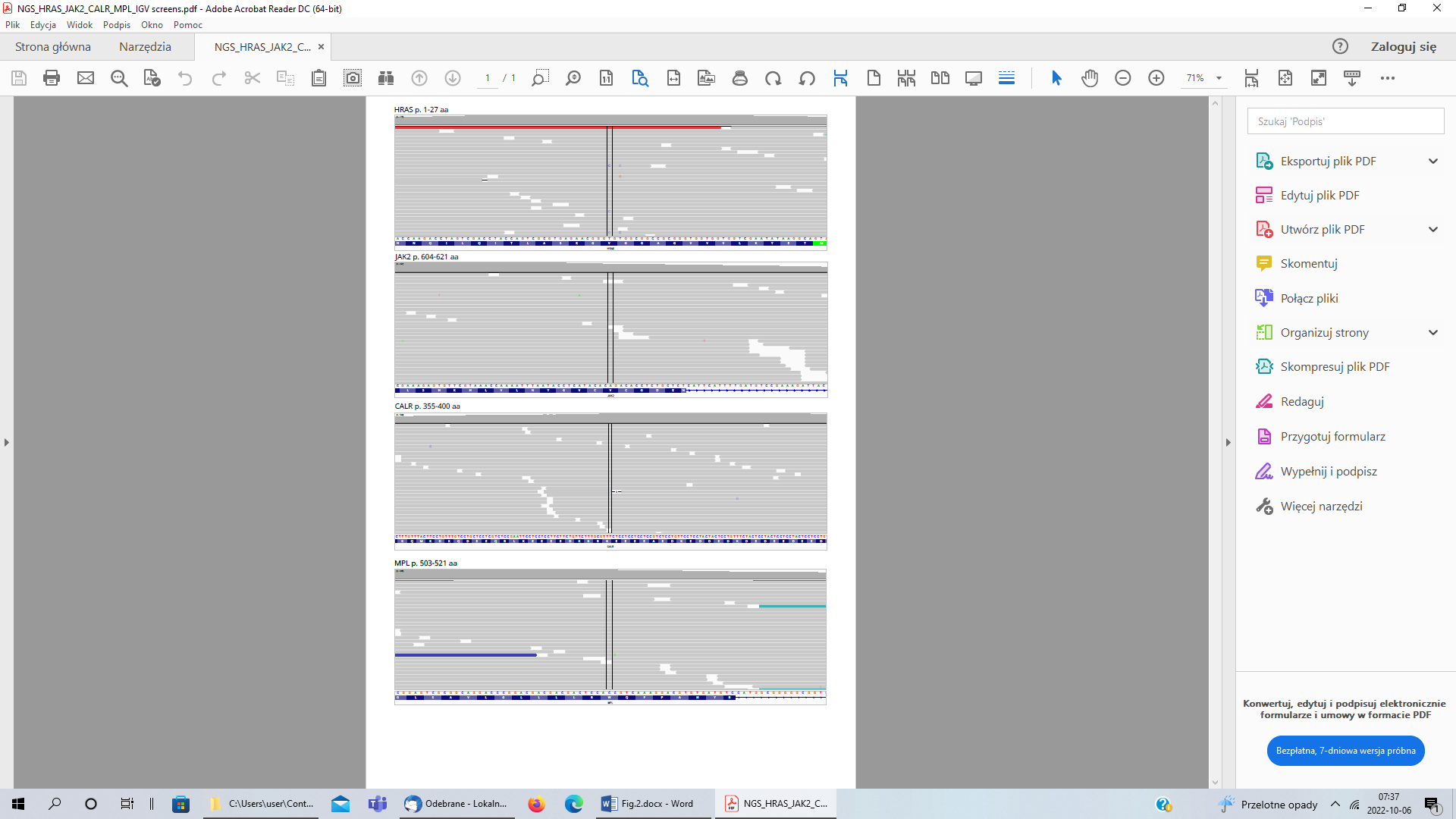


Abbreviations:

BM – bone marrow cells derived DNA, PB- peripheral blood leukocyte derived DNA, CALR - calreticulin gene, HRAS- the Harvey rat sarcoma gene, JAK2- Janus tyrosine kinase 2 gene, MPL- thrombopoietin receptor gene. The nucleotide sequence of substitution place is indicated by green line.
