# Supplementary material for: HRAS mutation positive multiple myeloma in the type 2 CALR mutation positive essential thrombocythemia: A case report
Source: J Cell Mol Med. 2023 Jan 5;27(2):299–303. doi: 10.1111/jcmm.17647 (PMC9843526; doi:10.1111/jcmm.17647)
Supplement: Supplementary file 2 — TableS1 [file JCMM-27-299-s001.docx]

Supplementary Table 1. Analysis of the co-occurrence of CALR gene and RAS (KRAS, NRAS, HRAS) genes mutations according to The Cancer Genome Atlas (TCGA) database, including datasets of >10000 samples of 33 cancer types.

|  | cacner type | nonsynonymous mutations in CALR | driver CARL mutation type-1: del52, | driver CARL mutation type-2: ins5 | HRAS mutation co-occurring with mut. in CARL | NRAS mutation co-occurring with mut. in CARL | mutation in KRAS mutation co-occurring with mut. in CARL |
| --- | --- | --- | --- | --- | --- | --- | --- |
| CC | adrenal land adenomas and adenocarcinomas | 0 | 0 | 0 |  |  |  |
| BLCA | bladder transitional cell papillomas and carcinomas | 1 | 0 | 0 |  |  |  |
| BRCA | brest ductal and lobular neoplasms | 1 | 0 | 0 |  |  |  |
| CESC | cervix uteri squamous cell neoplasms | 5 | 0 | 0 |  |  |  |
| CHOL | liver and intrahepatic bile ducts adenomas and adenocarcinomas | 0 | 0 | 0 |  |  |  |
| COAD | colon adenomas and adenocarcinomas | 6 | 0 | 0 |  | 1 | 2 (2xG13D) |
| DLBC | lymphoid neoplasm diffuse large b-cell lymphoma | 0 | 0 | 0 |  |  |  |
| ESCA | esophagus squamous cell neoplasms | 1 | 0 | 0 |  |  |  |
| GBM | glioblastoma multiforme | 0 | 0 | 0 |  |  |  |
| HNSC | head and neck squamous cell neoplasms | 0 | 0 | 0 |  |  |  |
| KICH | kidney adenomas and adenocarcinomas | 0 | 0 | 0 |  |  |  |
| KIRC | kidney adenomas and adenocarcinomas | 0 | 0 | 0 |  |  |  |
| KIRP | kidney adenomas and adenocarcinomas | 1 | 0 | 0 |  |  |  |
| LAML | acute myeloid leukemia | 3 | 0 | 0 |  | 1 (1xG13D) |  |
| LGG | low grade glioma | 2 | 0 | 0 |  |  |  |
| LIHC | liver and intrahepatic bile ducts adenomas and adenocarcinomas | 4 | 0 | 0 |  |  |  |
| LUAD | lung adenocaricnomas | 2 | 0 | 0 |  |  | 1 |
| LUSC | lung squamous cell neoplasms | 3 | 0 | 0 |  |  |  |
| MESO | mesothelioma | 0 | 0 | 0 |  |  |  |
| OV | ovary cystic, mucinous and serous neoplasms | 1 | 0 | 0 |  |  |  |
| PAAD | pancreas ductal and lobular neoplasms | 0 | 0 | 0 |  |  |  |
| PCPG | pheochromocytoma and paraganglioma | 0 | 0 | 0 |  |  |  |
| PRAD | adrenal gland adenomas and adenocarcinomas | 1 | 0 | 0 |  |  |  |
| READ | rectum adenomas and adenocarcinomas | 0 | 0 | 0 |  |  |  |
| SARC | sarcoma | 0 | 0 | 0 |  |  |  |
| SKCM | skin cutaneous melanoma | 5 | 0 | 0 |  |  |  |
| STAD | stomach adenomas and adenocarcinomas | 3 | 0 | 0 |  |  |  |
| TGCT | testicular germ cell tumors | 0 | 0 | 0 |  |  |  |
| THCA | thyroid gland adenomas and adenocarcinomas | 0 | 0 | 0 |  |  |  |
| THYM | thymic epithelial neoplasms | 0 | 0 | 0 |  |  |  |
| UCEC | uterine corpus endometrial carcinoma | 16 | 0 | 0 | 2 | 4 | 3 |
| UCS | uterine carcinosarcoma | 1 | 0 | 0 |  |  |  |
| UVM | eye and adnexa nevi and melanomas | 0 | 0 | 0 |  |  |  |
| PAN-C | **total** | 56 | 0 | 0 |  |  |  |
